# 3D palatal superimposition in adolescent orthodontic patients treated without extractions: method validation

**DOI:** 10.1007/s00784-025-06350-0

**Published:** 2025-05-05

**Authors:** Caroline Heni, Eva Henninger, Pawel Pazera, Georgios Vasilakos, Nikolaos Gkantidis

**Affiliations:** 1 Department of Orthodontics and Dentofacial Orthopedics, School of Dental Medicine, University of Bern, Bern, CH-3010 Switzerland; 2 Private Practice, Frankfurter Strasse 610, 51145 Cologne, Germany

**Keywords:** Three-dimensional superimposition, Palatal rugae, Digital dental model, Growing patients, Orthodontic treatment

## Abstract

**Objectives:**

This study investigated the morphological stability, trueness and precision of four palatal areas (A: medial 2/3 of third ruga and 5 mm posteriorly; B: A + additional 5 mm posteriorly; C: A + additional 6 mm wide stripe on midpalatal suture; D: almost whole palate) used for 3D superimposition of serial maxillary dental models in growing orthodontic patients treated without extractions.

**Materials and methods:**

A retrospective sample of 25 growing patients with pre- (T0) and post- (T1) treatment 3D dental models was used. Morphological stability was assessed using color-coded distance maps generated from superimposed T0 and T1 models. Trueness was determined by measuring the Mean Absolute Distances (MAD) within Area A between T0 and T1 models after superimposition. Precision was evaluated through the assessment of T0-T1 changes in three preselected teeth.

**Results:**

Treatment and growth modified the palatal surface morphology at the 0.5 mm level. Trueness remained below 0.3 mm in most cases for Areas A, B, and C, with a reduced level observed for Area D. The precision outcome was significantly affected with respect to tooth torque and rotations. Area D showed the best precision, followed by B, C, and A. Area B showed the best agreement to Area C. The overall assessment favored Area B.

**Conclusion:**

Considering overall performance, area B is recommended as the area of choice for superimposing pre-to post-orthodontic treatment maxillary dental models of growing individuals treated without extractions.

**Clinical relevance:**

The suggested palatal superimposition method is broadly applicable and ensures reliable assessment of morphological changes through the superimposition of serial maxillary dental models.

**Supplementary Information:**

The online version contains supplementary material available at 10.1007/s00784-025-06350-0.

## Introduction

In many fields of medicine, clinicians and researchers have long been interested in assessing changes in craniofacial morphology over time and particularly in analyzing treatment effects. In orthodontics, several approaches have been applied over the years to evaluate the effects of orthodontic interventions and to assess growth, ranging from conventional 2D radiograph superimpositions [1, 2] to more recently developed 3D techniques [3–5]. The main 3D imaging modalities to assess craniofacial morphology include intraoral 3D surface models [4, 6], 3D radiographs [7] and 3D facial photographs [8, 9] or videos [10].

The 3D superimposition of digital dental models enables a risk-free, precise evaluation of several outcomes including, tooth movement [11] and dental [12] or periodontal tissue changes [13]. However, the establishment of any superimposition technique for regular use prerequisites proper validation, including accuracy, precision, applicability and reproducibility testing.

The palatal rugae area has been since long considered as a stable superimposition reference to best-fit approximate serial dental models [11, 14]. However, morphological changes are expected to occur in the palate during growth, with or without treatment [14–18]. Tooth movement occurring due to fixed or removable orthodontic appliances may impact palatal morphology [15], potentially compromising the 3D superimposition outcomes or leading to misinterpretations. Several studies suggest that the third ruga is considered as relatively stable structure, also during growth [14, 17] and treatment [4, 9, 18, 19]. However, there is still significant heterogeneity in study types, patient samples, model acquisition techniques, treatment types, superimposition methods, as well as in outcome assessment and statistical analysis methods. Many studies to date have tested differences in mean values but have not examined differences between individual measurements, which is necessary to establish a method as appropriate for application in individual cases [20, 21].

A few years ago, *Vasilakos et al.* [6]. tested five different superimposition reference areas, which were previously applied in other studies, on models obtained before and after an interceptive treatment in early mixed dentition. This study clearly demonstrated that the reference area used for palatal superimposition has an important influence on superimposition outcome in growing patients. The study concluded that the superimposition on the medial part of the third ruga and a small area dorsal to it provides accurate results. The incorporation of other palatal areas was not recommended. However, an increased superimposition reference area has been shown to be beneficial in terms of robustness to artifacts or local surface changes [22]. It is important to note that the study by *Vasilakos et al.* [6] focused on young patients undergoing interceptive treatment in the early mixed dentition, which involved limited tooth movement. There is still a lack of solid evidence in the field regarding regular orthodontic patients, namely adolescents subjected to non-extraction treatment with fixed orthodontic appliances. Thus, the aim of our study was to assess the performance of different palatal areas in terms of accuracy of surface-based superimposition of serial maxillary 3D dental models derived from adolescents who received non-extraction orthodontic treatment with fixed appliances. The null hypothesis was that the superimposition reference area selection on the palate does not affect the trueness and precision outcomes in terms of tooth movement assessment.

## Materials and methods

### Sample

The sample consisted of pre- (T0) and post-orthodontic (T1) treatment 3D dental models of 25 growing patients (7 males, 18 females; mean age at treatment start: 12.2 ± 0.9, range: 10.9–13.5 years old; mean T0-T1 period: 2.15 ± 0.7, range: 1.3–3.7 years), retrospectively selected from the archives of the Department of Orthodontics and Dentofacial Orthopedics of the University of Bern, Switzerland and two private practices in Switzerland. The following inclusion criteria were applied: (a) non-extraction orthodontic treatment, (b) age at treatment start between 10 and 13 years, (c) treatment duration between 1.5 and 3.5 years, (d) minimum 1 year after the end of any previous orthodontic treatment at T0, (e) buccal fixed appliances in both jaws (at least 6–6), (f) Class I and less than ½ cusp Class II at T0, (g) maximum 6 months from initial records to treatment start, (h) early permanent dentition at pre-treatment (T0; 1st molar to 1st molar fully erupted, except from 2nd premolars), (i) clinically acceptable occlusion at T1 (< 1/4 deviation from Class I), (j) good quality 3D dental models and cephalometric radiographs at pre- and post-treatment, (k) no congenital anomalies, syndromes or systemic diseases that could affect craniofacial morphology or tooth movement. A description of the applied exclusion criteria is provided in Table 1.

**Table 1:** Exclusion criteria applied during the patient sample selection

Exclusion Criteria
• No appliance at the palate at least 1 year prior to T0 and during treatment
• No impacted or ectopically erupted canines
• No extreme GoGnSN (NSL/ML) angle (± 2SD from mean) at T0
• No extreme facial contour angle (± 2SD from mean) at T0
• No extreme ANB angle (7 > ANB > 1) at T0
• No extreme malocclusions (1 < OB < 6 mm; 1 < OJ < 6 mm; 1 < Crowding < 6 mm)
• No severe posterior crossbites at T0 (> 3.5 mm total transversal discrepancy)
• No large asymmetries (visual inspection)
• No missing teeth from 2nd molar to 2nd molar
• No active expansion of dental arches (e.g. Hyrax or quad helix)
• No appliance in contact with the palate (e.g. activator, mini screws, Nance)

A sample size calculation before the start of the study was not performed due lack of pre-existing data. Based on the authors’ experience and empirical evidence, we aimed to collect a sample of 25 patients to ensure both adequate statistical power and an appropriate assessment of variation. This sample size was deemed sufficient to achieve a valid representation of the studied population [4, 23–25].

### 3D model acquisition

The 3D digital dental models originated from conventional dental casts obtained through alginate impressions that were poured with orthodontic plaster on the same day. Each stone cast was then scanned with a high accuracy laboratory 3D surface scanner (Stripe light/LED illumination, Cendres + Métaux SA, CH; accuracy < 20 μm). 3D surface models (STL) were generated (100,000-350,000 vertices per model) for every patient at T0 and T1 for further processing.

### Superimposition workflow

**Fig. 1:**
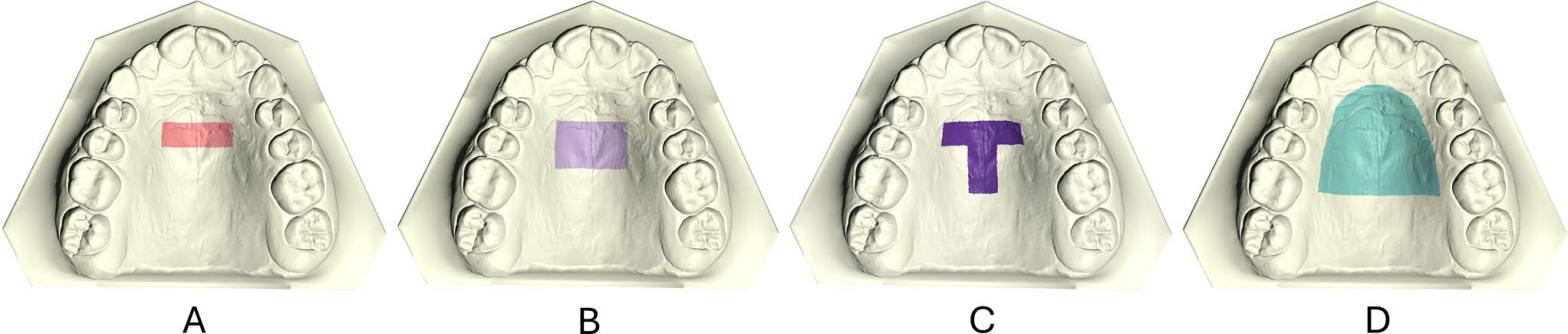
Palatal areas used as superimposition references, depicted with different colors on the maxillary models. **A**: Area A; **B**: Area B; **C**: Area C; **D**: Area D

3D pre-treatment (T0) and post-treatment (T1) dental models were imported in Viewbox 4 software (version 4.1.0.1 BETA, dHAL, Software, Kifissia, Greece), where four different superimposition reference areas were selected on the T0 models for testing (Fig. 1). These were:

1. Area A: area of the palate limited anteriorly by the medial 2/3 of the third ruga and laterally by two lines paralleled to the midpalatal suture and extending posteriorly 5 mm from the third ruga.
2. Area B: Area A extended posteriorly additional 5 mm.
3. Area C: Area A with an additional 6 mm wide stripe on the midpalatal suture, extending posteriorly to the level of a line connecting the lingual grooves of the 1st permanent molars at the gingival level.
4. Area D: almost to the whole palate, delimited by a line 5 mm distant from all gingival margins and extending posteriorly until a line connecting the lingual grooves of the 1st permanent molars at the gingival level.

The models of each T0-T1 pair were best-fit approximated using each of the aforementioned references areas, starting always from the original model position. This was performed by applying the software’s iterative closest point algorithm (ICP) under the following settings [4, 13]: 100% estimated overlap of meshes, matching point to plane, exact nearest neighbor search, 100% point sampling, 50 iterations.

**Fig. 2:**
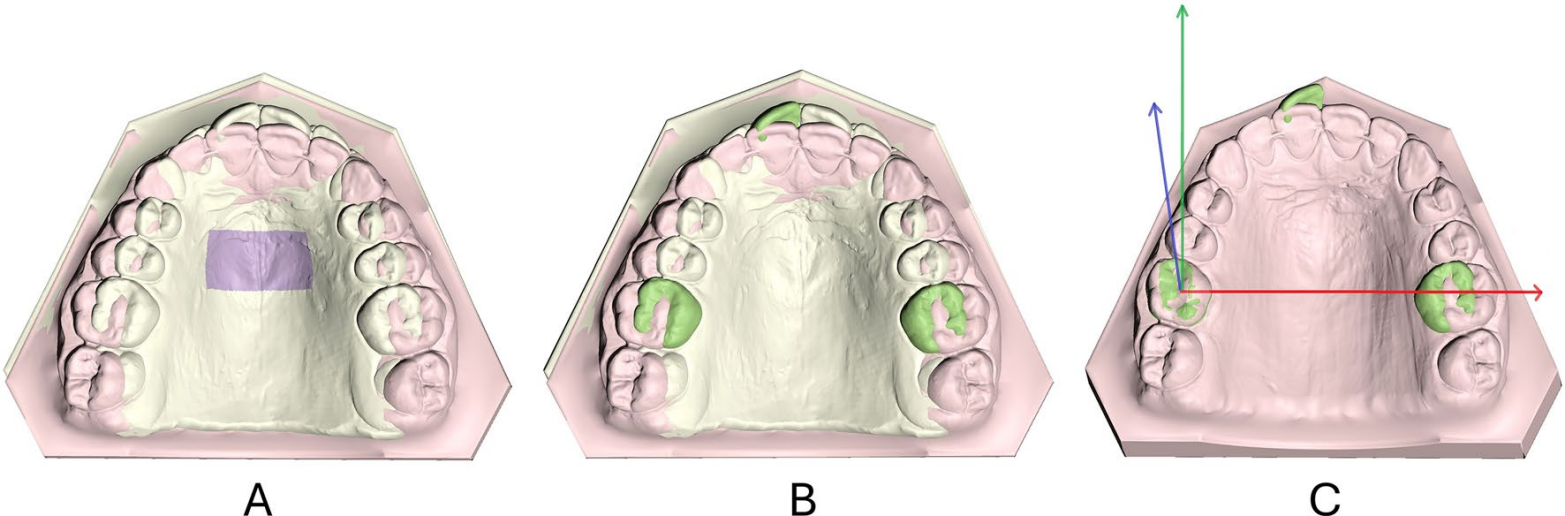
Workflow of tooth movement assessment following palatal superimposition. **A**. Superimposition of pre- (beige color) and post-treatment (pink color) maxillary models on Area B (purple color). **B**. Selection of the tooth crowns of interest for tooth movement assessment (green color). **C**. Superimposition of pre- to post-treatment right maxillary first molar crown and recording of the required translations and rotations along the depicted axes, with their origin placed at the crown’s centroid. The same axes orientation was used for all measured teeth. The spatial changes of the teeth have been recorded according to patient’s anatomical reference as follows. Tooth translations. X-axis (red color): lateral movement, positive: left; Y-axis (green color): anteroposterior movement, positive: anterior; Z-axis (blue color): vertical movement, positive: down/occlusal. Tooth rotations. X-axis (red color): posteriorly tipping/anteriorly torque, positive: distal tipping/palatal crown torque; Y-axis (green color): posteriorly torque/anteriorly angulation, positive: palatal crown torque/mesial crown angulation; Z-axis (blue color): rotation (posteriorly and anteriorly), positive: mesial out rotation

Tooth movement was calculated for one maxillary central incisor and the two contralateral first permanent molars. These were selected once on the T0 models and used for all tests. After each palatal superimposition, the preselected teeth crowns at T0 were superimposed individually on the respective ones at T1, using the aforementioned settings, but with 80% estimated overlap of meshes [26, 27]. As described in detail in *Vasilakos et al.* [6], this way, the positional changes of each tooth crown from T0 to T1 were recorded in all 3 dimensions, with the origin of the axis positioned at each crown centroid and the axis of movement parallel to the midline palatal suture and vertical to the occlusion plane (Fig. 2). The centroid of each crown was defined as the arithmetic mean position of all segmented crown surface mesh vertices [28] and was automatically generated by the software. The reliability of this method is directly dependent on the accuracy of the clinical crown surface segmentation. Previous studies have demonstrated that this approach achieves a high level of reliability [6]. Additionally, since this process is automated and does not rely on manual landmark placement, the potential for intra- or inter-examiner variability is minimized.

### Assessed outcomes

The morphological stability/congruence of the different palatal areas was assessed visually through color-coded distance maps, illustrating the distances between superimposed pairs of T0-T1 models at each superimposition reference area. This was quantified through the calculation of the Mean Absolute Distances (MAD) between closest points of the superimposed reference areas.

To evaluate the trueness of the superimposition on areas A, B, C and D, we assessed the congruence of the two models in area A, by measuring the MAD of each mesh vertex of the T0 model to the T1 surface. Only the vertices within area A, the supposed anatomically most stable area [4, 6, 14, 19, 29, 30], were considered in the computation, irrespective of the area used for superimposition.

The precision of the superimposition areas was evaluated through the assessment of positional changes of three teeth of interest (one central incisor and two first molars) between two repeated superimpositions. The agreement between different superimpositions was assessed on the same outcomes.

A 0–3 grading of the performance of each area in each tested outcome, concluding to an overall assessment for each area, was performed by the first and the last author, with 0 indicating minimum and 3 optimal performance.

### Method error

All palatal superimpositions on each reference area were performed twice by the first author, with an interval of two weeks, to test reproducibility. A previous study showed no substantial differences between repeated selections of the superimposition reference areas or tooth crowns [6]. Therefore, all superimpositions were repeated using the same surface area selections and with the T0 model held constant as a reference. The MAD between the resulting T1 models, following repeated superimpositions on the constant T0 model, at each reference area, was calculated to verify method reproducibility. Zero MAD would indicate perfect reproducibility. The maximum absolute differences in MAD values between repeated superimpositions on areas A, B, C, and D, on the respective areas, were 0.011, 0.010, 0.018, and 0.001 mm, indicating perfect reproducibility.

The assessment of the overall performance of each superimposition reference area was performed by two additional assessors with experience in 3D digital imaging and superimpositions, one co-author (P.P.) and a non-author, independently. Both provided similar outcomes to the original assessment.

### Statistical analysis

Statistical analysis was carried out by using the SPSS Software (IBM SPSS Statistics for Windows, Version 28.0. Armonk, NY: IBM Corp.).

Raw data were tested for normality through the Kolmogorov-Smirnov and Shapiro-Wilko tests and did not have a normal distribution in certain cases. Thus, non-parametric statistics were applied.

In all cases, a two-sided significance test was carried out at an alpha level of 0.05. A Bonferroni correction was applied for pairwise *a posteriori* multiple comparison tests, to reduce the possibility of false positive results.

To overcome potential limitations of methods that are solely based on mean/median comparisons described above, the extent and variation of all individual measurements is shown in box plots and was considered in the assessments.

## Results

**Fig. 3:**
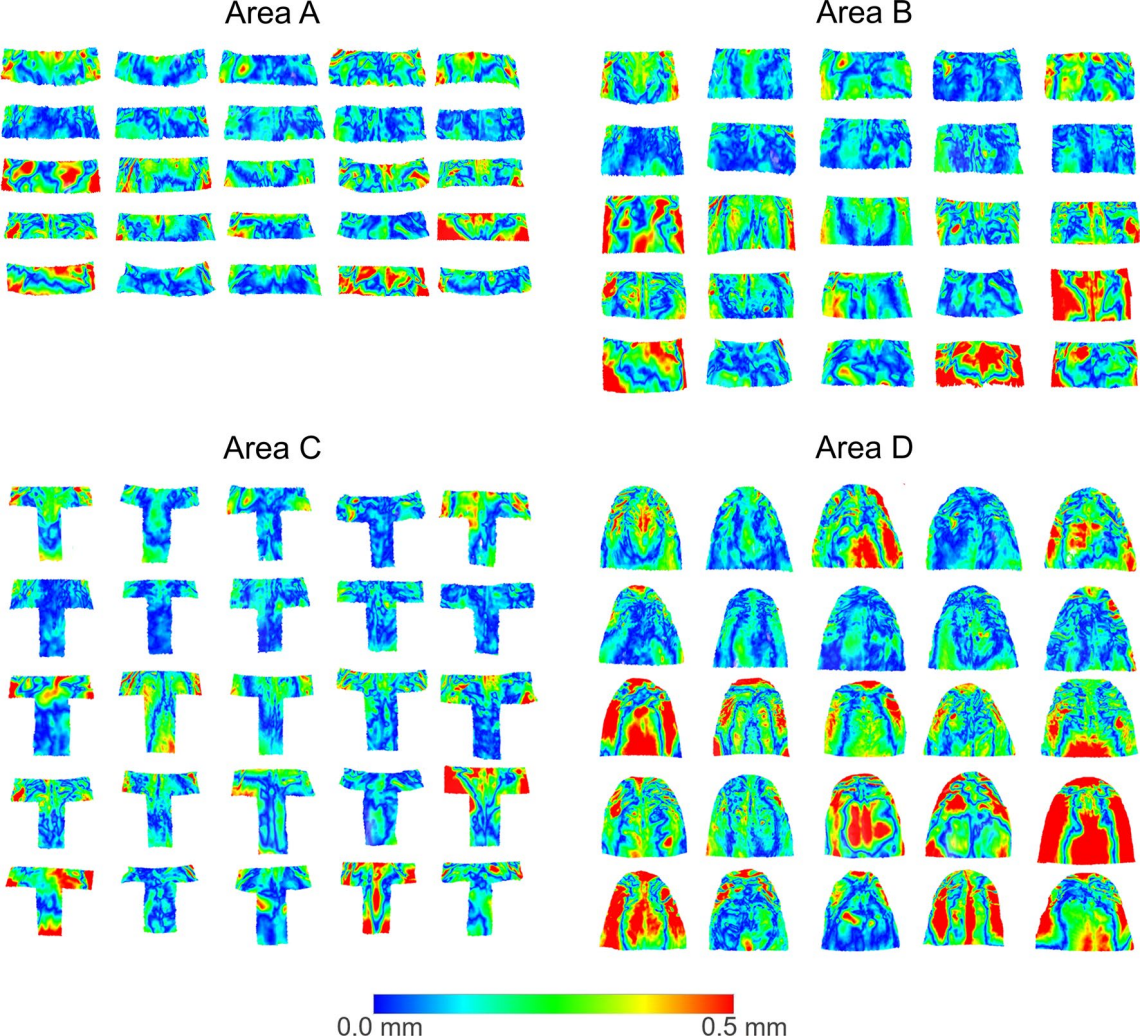
Color-coded distance maps, illustrating the distances between superimposed pairs of T0/T1 models at each superimposition reference area

**Fig. 4:**
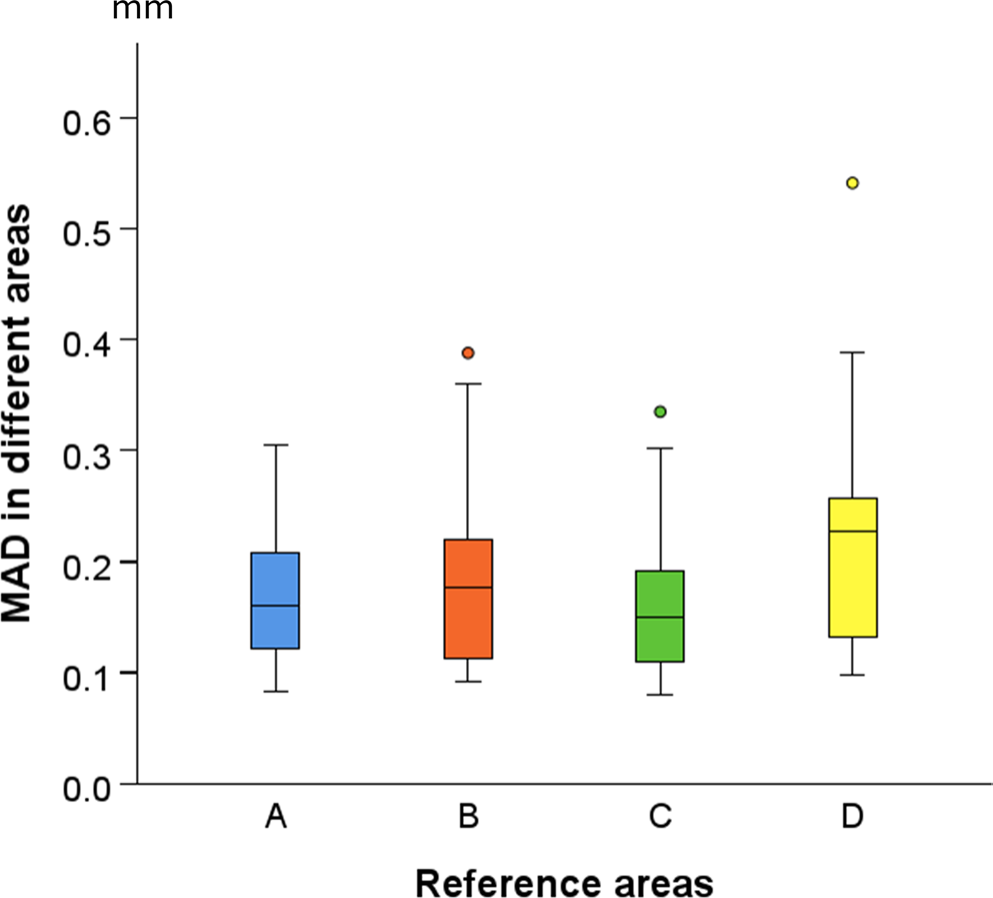
Box plots illustrating the Mean Absolute Distance (MAD) for each reference area following the T0/T1 model superimposition

The visual assessment of the color-coded distance maps, between superimposed pairs of T0-T1 models, at each superimposition reference area, showed that in several cases, the treatment and growth effects modified the palatal surface morphology at the 0.5 mm level (Fig. 3). After each superimposition, the median (IQR: interquartile range) MADs between Areas A, B, C, and D were 0.160 (0.095), 0.177 (0.113), 0.151 (0.092), and 0.227 (0.134) mm, respectively (Kruskal Wallis test: *p* = 0.058; Bonferroni adjusted pairwise tests: *p* > 0.05) (Fig. 4).

**Fig. 5:**
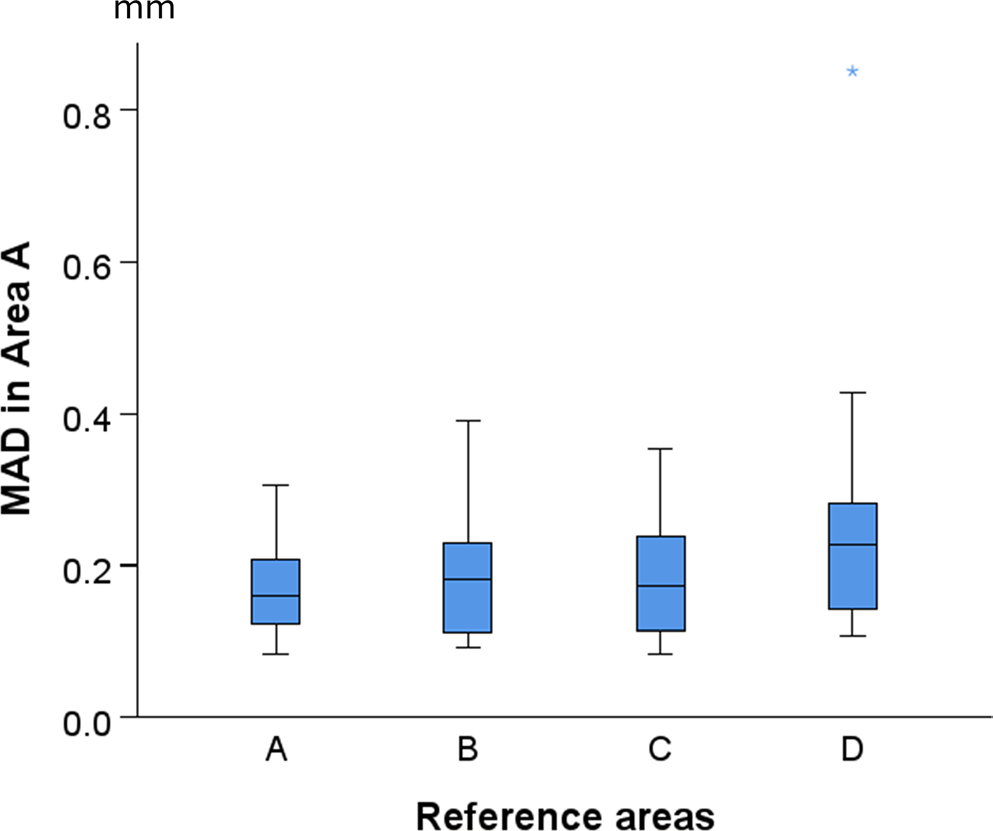
Box plots illustrating the Mean Absolute Distance (MAD) in area A across all superimposition reference areas

The superimposition reference area selection in the palate did not affect the trueness outcomes, which ranged between 0.083 and 0.851 mm, but remained consistently below 0.4 mm for Areas A-C. After each superimposition on Areas A, B, C, and D, the median (IQR) MADs in Area A between superimposed T0-T1 models were 0.160 (0.095), 0.182 (0.124), 0.174 (0.132), and 0.227 (0.170) mm, respectively (Kruskal Wallis test: *p* = 0.100; Bonferroni adjusted pairwise tests: *p* > 0.05) (Fig. 5).

**Fig. 6:**
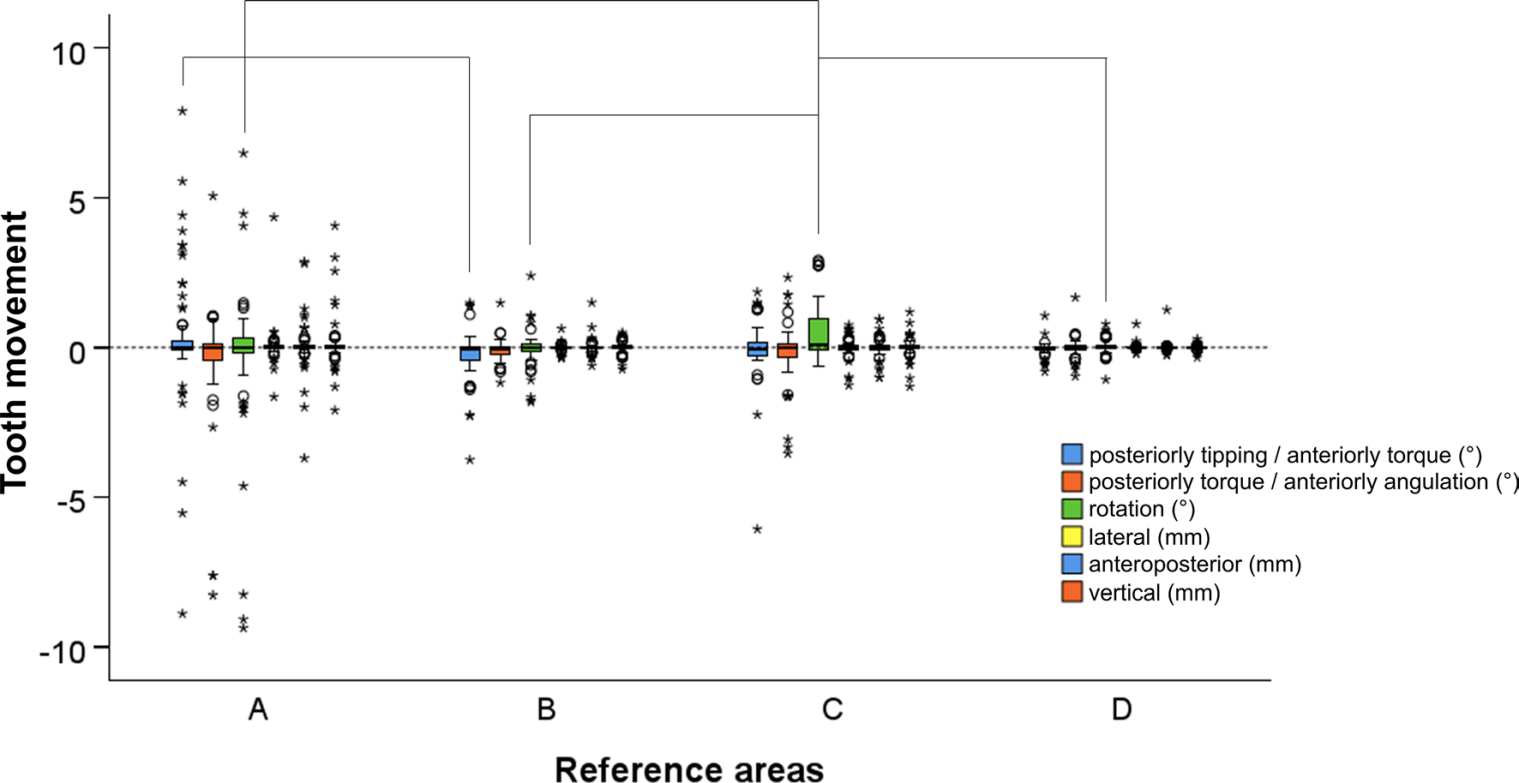
Box plots showing the precision in different tooth movements. The black horizontal lines indicate statistically significant differences between the connected variables at the 0.05 level (Bonferroni adjusted; Dunn’s test)

The superimposition reference area selection significantly affected the precision outcomes, in terms of tooth movement assessment, for two variables, namely X-rotation (posteriorly tipping/anteriorly torque) and Z-rotation (in/out rotations) (Kruskal-Wallis test: *p* = 0.005 and *p* = 0.003, respectively) (Fig. 6). Overall, Area D showed the best precision (median: 0.00, IQR: 0.04 mm or °), followed by Area B (median: 0.00, IQR: 0.14 mm or °), whereas Areas C (median: 0.00, IQR: 0.23 mm or °) and A (median: 0.00, IQR: 0.23 mm or °) showed similar results (Fig. 6). Visualization of scatter dot plots depicting the precision measurements for each individual tooth yielded similar outcomes, with no evidence of noticeable differences among the different teeth (Supplementary Figs. 1 and 2).

**Fig. 7:**
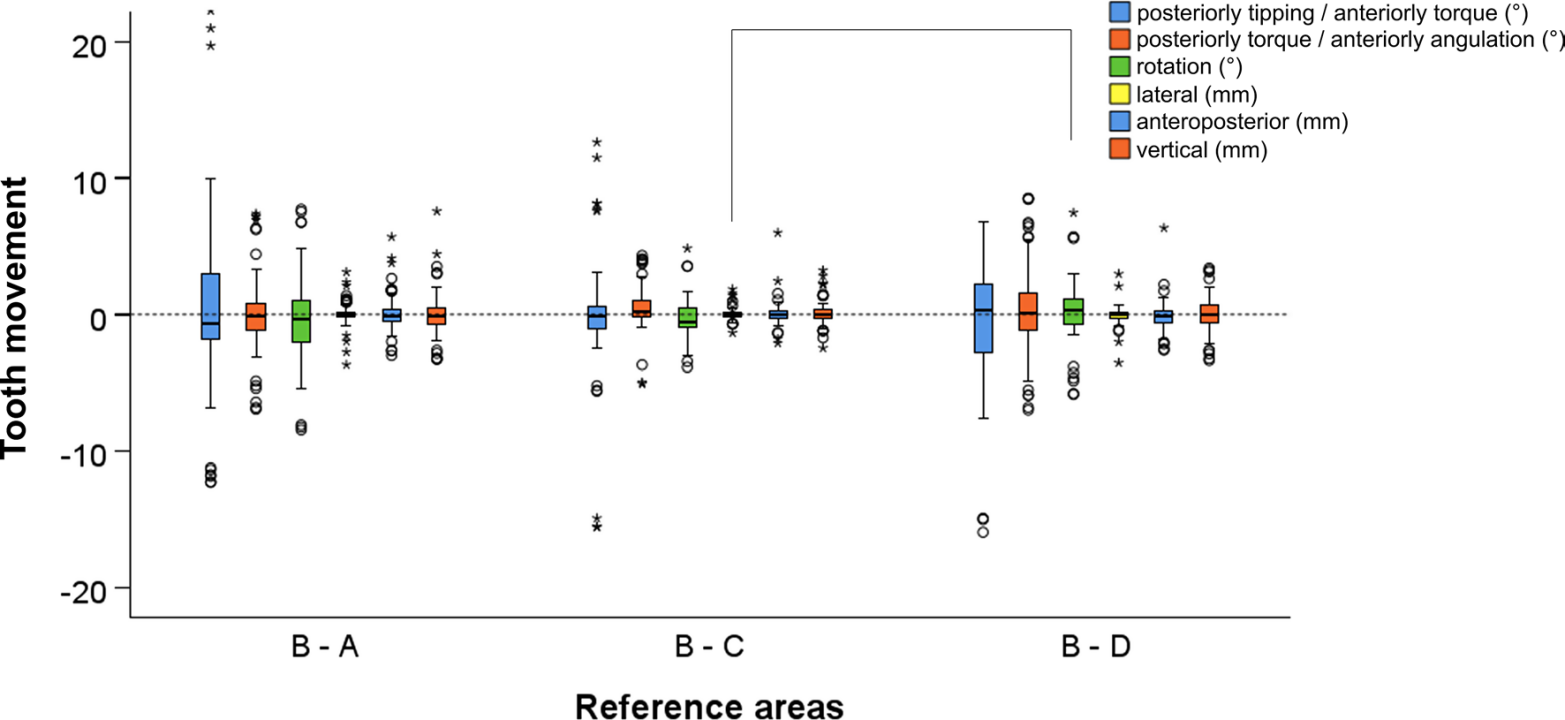
Box plots showing the agreement in different tooth movements. The black horizontal line indicate statistically significant differences between the connected variables at the 0.05 level (Bonferroni adjusted; Dunn’s test)

The agreement between the superimposition areas was evaluated through the assessment of differences in the detected positional changes at the three teeth of interest. Due to the previous outcomes, Area B was compared to the performance of the other areas and only one significant difference was detected (Fig. 7). Overall, Area B showed the best agreement with Area C (median: -0.03, IQR: 0.80 mm or °), followed by Areas A (median: -0.11, IQR: 1.53 mm or °) and D (median: 0.02, IQR: 1.38 mm or °), which showed similar results (Fig. 7).

### Summary assessment

- **Area A** shows comparable trueness to areas B and C, but low precision, especially compared to areas B and D. Area A also shows reduced agreement on tooth movement assessment with area B.
- **Area B** shows comparable trueness to areas A and C, but higher precision in tooth movement assessment. It also shows high agreement to area C, but lower agreement to areas A and D. The visualization of the color-coded distance maps between superimposed T0-T1 models does not indicate higher differences at the surface extension from area A as compared to area A.
- **Area C** shows comparable trueness to areas A and B, but is not as precise as area B, with which it shows high agreement.
- **Area D** shows the highest precision, but has reduced trueness compared to the other three areas and less agreement with area B in tooth movement assessment.

Table 2 provides an overview of the comparative analysis of all tested outcomes, illustrating the favorable performance of Area B.

**Table 2:** Comparative analysis of the four superimposition reference areas across all tested outcomes. Each outcome was scored on a scale from 0 to 3, with 0 representing the lowest performance (minimum score) and 3 indicating the highest performance (optimal score)

	Area A	Area B	Area C	Area D
Reference area matching	3	3	3	1
Trueness	3	3	3	2
Precision	0	2	1	3
**Total**	**6**	**8**	**7**	**6**
Agreement to Area B	1	-	3	2

## Discussion

The aim of the study was to evaluate four different reference areas for palatal surface superimposition in terms of their trueness, precision and stability. Area A was considered the standard reference, since previous literature reported this area (medial two-thirds of the third ruga and the area 5 mm dorsal to them) as being the most stable over time [6, 11, 30, 31]. Our findings indicated that Area B, which includes Area A with an additional 5 mm extension posteriorly, performed best overall in a typical sample of growing orthodontic patients, treated without extractions. Area C followed closely, showing the greatest agreement to Area B. Area A was not proved suitable due to reduced reproducibility attributed to its small size [22, 32]. A previous study [4] on a similar sample suggested that the second and third rugae can be included in the superimposition reference area, as they were both subjected to similar changes over time [4]. This enlarges the reference area, reducing the impact of artefacts and yielding more robust results [22, 32]. In accordance with previous studies [22, 32] this was evident in the present *precision* outcomes, evaluated through the assessment of positional changes of three teeth. Area D encompassing almost the entire palatal surface showed the highest precision.

Apart from local changes in palatal surface morphology that can affect the superimposition outcome, artefacts are likely to be present in any type of models, and therefore, it is important to consider this factor [22]. Artefacts in plaster models can result from the impression technique, the presence of saliva, or the stone pouring process. Artefacts in plaster models are usually small, bubble-type structures [4, 22, 33], which are likely to appear in morphologically complex areas such as the rugae. For intraoral digital models, artefacts are most likely to occur in movable structures [34], but also elsewhere due to saliva and moisture, patient movement, highly reflective surfaces, such as dental restorations or metal appliances, operator technique, skill and experience, machine hardware and software performance, or interferences from adjacent structures [22, 35–37].

It has been shown that a small alteration in surface morphology of a digital model can considerably affect tooth movement assessment through palatal surface superimposition [22]. The larger the superimposition reference area, the more tolerance for local morphological deviations on the superimposed areas (Area D). On the contrary, a small area such as Area A shows low precision due to its susceptibility to artefacts or morphological alterations.

In this context, it makes perfect sense that Area B (Area A extended posteriorly 5 mm) showed the second best precision, due to its larger extent. In terms of precision, we can conclude that the larger the area, the greater the tolerance for morphological deviations between the superimposed palatal surfaces. The present findings, consistent with previous studies, indicate that very small superimposition reference areas exhibit reduced precision in the presence of even limited morphological deviations between the superimposed surfaces. Moreover, beyond a certain point, further enlargement of the reference area results in only minimal improvements in precision [3, 22, 32].

Validating a superimposition area requires fulfilling several conditions, with both precision and trueness being key factors. Historically, metallic implants were inserted in various jaw locations as gold-standard references for growth assessment [1]. However, due to ethical concerns, this approach is no longer feasible. More recently, researchers have used unloaded orthodontic mini screws inserted in interradicular maxillary spaces [31] or loaded mini screws placed in the palate [16], for similar purposes. However, these studies focused on non-growing orthodontic patients. Our study investigated adolescent patients with mild to moderate malocclusions treated without extractions, in whom skeletal anchorage is not required. Furthermore, mini screw placement in the palatal region could influence the tested area. To evaluate the trueness of each superimposition technique, we assessed the congruence between models in area A by measuring the MAD of each T0 mesh vertex from the T1 model. The vertices within area A—an anatomically stable region during growth and orthodontic treatment [4, 6, 14, 19, 29, 30]—were considered in the computation, regardless of the superimposition area used. Trueness was comparable for Areas A, B and C, whereas Area D scored the worst, supposedly due to its proportionally larger surface extent difference from area A.

A recent study analyzed the palatal rugae morphological stability in adult orthodontic patients [29] suggesting that especially the first ruga morphology is influenced by orthodontic treatment with extractions. This is in line with our previous suggestion for growing orthodontic patients treated without extractions that the second and third rugae can be used as superimposition references, but not the first, which is affected by anterior tooth movement [4]. Other studies have also indicated the lack of stability for the first ruga [4, 18, 38]. The closer a ruga is to the teeth the larger the anticipated effect of tooth movement on palatal structures.

Morphological changes in the palatal rugae area also occur after palatal expansion for transverse corrections [15, 39]. The greatest effects were observed in the third ruga [24], followed by the second and first rugae [39]. This reflects the expected relapse pattern following active expansion. Based on these findings, studies have recommended using the first ruga as the preferred area for superimposition after palatal expansion, despite the minimal or negligible changes observed beyond the first year [15]. However, this recommendation overlooks the impact of natural palatal growth in the absence of expansion and the effects of orthodontic tooth movement, both of which suggest that the second and third rugae may be more reliable for superimposition [4, 14, 17].

Most available studies so far have focused on extraction cases. The samples vary from our study, as most of the studies assessed non-growing patients [16, 29, 38]. However, all studies recommended the third ruga as an appropriate superimposition reference area. Jang et al. [16] suggested the medial points of the third ruga, Bailey et al. [38] suggested the medial and lateral points of the third ruga and Zhao et al. [29] suggested the second and third rugae. Pazera & Gkantidis [4] also suggested the second and third rugae after testing a growing, non-extraction orthodontic patient sample. The aforementioned studies tested the palatal rugae morphological stability based on landmark distances and did not evaluate 3D surface superimposition outcomes, as in the present study. However, their outcomes support our conclusion that Area B should be the area of choice, especially for extraction cases with considerable anteroposterior tooth movement.

As previously suggested [4], in non-extraction cases, the second ruga can be alternatively included in the superposition reference area to add robustness to morphological deviations between the superimposed surfaces. The addition of the second ruga area to Area A, creates an area of similar extent to Area B, and thus, of similar robustness to morphological deviations between the superimposed surfaces. However, the reference area in extraction cases needs to be anteroposteriorly further from the teeth to be less affected by considerable tooth movement. Bailey et al. [38] showed that space closure through retraction of maxillary anterior teeth causes sagittal changes at the lateral and medial points of the second ruga. This has also been supported by Jang et al. [16] and van der Linden [40], indicating that the closer the rugae are to the site of tooth movement, the less stable they become.

Through the analysis of a variety of outcomes, i.e. the congruence of the reference area, precision, and trueness, this study provided a thorough analysis of the performance of different superimposition reference areas. Using this approach, a valid method for superimposition in growing, non-extraction orthodontic patients has been developed and by extrapolating the results, along with information from the existing literature, this method should be also suitable for extraction cases. The study also showed that the superimposition reference area selection impacts the outcomes, despite that area A was included in all other areas. This has been also shown previously for dental models from a younger patient sample [6] as well as for the superimposition of 3D facial photos of growing patients [9].

The findings of this study highlight the clinical value of serial maxillary dental model superimposition as a practical, rapid, risk-free, and reliable 3D tool for assessing intraoral morphological changes over time [11]. The oral environment undergoes continuous changes due to growth, treatment, and pathology, affecting tooth form, periodontal tissue morphology, and tooth position. Superimposing serial dental models on a stable structure provides reliable insights into various clinical outcomes, including orthodontic tooth movement assessment, treatment progress monitoring, evaluation of tooth positional stability, periodontal tissue changes, tooth wear analysis, early diagnosis of ankylosis through the detection of unaltered tooth position after force application, and the development of dental arch asymmetries relative to the palate [8, 12, 13, 24, 26, 41, 42].

A limitation of this study is that the sample included growing patients without severe malocclusions that were treated without extractions. This narrows the generalizability of our findings, although they were assessed in a constructive way together with findings from the literature. On the other hand, we validated a surface superimposition method for the most targeted patient group for orthodontists, using a robust sample.

## Conclusion

The present study thoroughly tested the morphological stability, trueness, and precision of four palatal surface areas for superimposing pre- to post-orthodontic treatment models of growing patients treated without extractions. Treatment and growth effects modified the palatal surface morphology usually within the 0.5 mm level. There were differences between areas in specific outcomes. Overall, the best performing reference area was Area B, which is limited anteriorly by the medial 2/3 of the third ruga and extends 10 mm posteriorly. This area is recommended for superimposing serial maxillary dental models of orthodontically treated growing patients to assess dentoalveolar changes.

## Electronic supplementary material

Below is the link to the electronic supplementary material.

Supplementary Material 1

## Data Availability

Data is provided within the manuscript or supplementary information files. Raw data can be provided from the corresponding author upon reasonable request.
