# Supplementary material for: 3D palatal superimposition in adolescent orthodontic patients treated without extractions: method validation
Source: Clin Oral Investig. 2025 May 5;29(5):285. doi: 10.1007/s00784-025-06350-0 (PMC12050233; doi:10.1007/s00784-025-06350-0)
Supplement: Supplementary file 1 — Supplementary Material 1 [file 784_2025_6350_MOESM1_ESM.pdf]

Clinical Oral Investigations

Supplementary material

3D palatal superimposition in adolescent orthodontic patients treated without extractions: method validation

Caroline Heni, Eva Henninger, Pawel Pazera, Georgios Vasilakos, Nikolaos Gkantidis

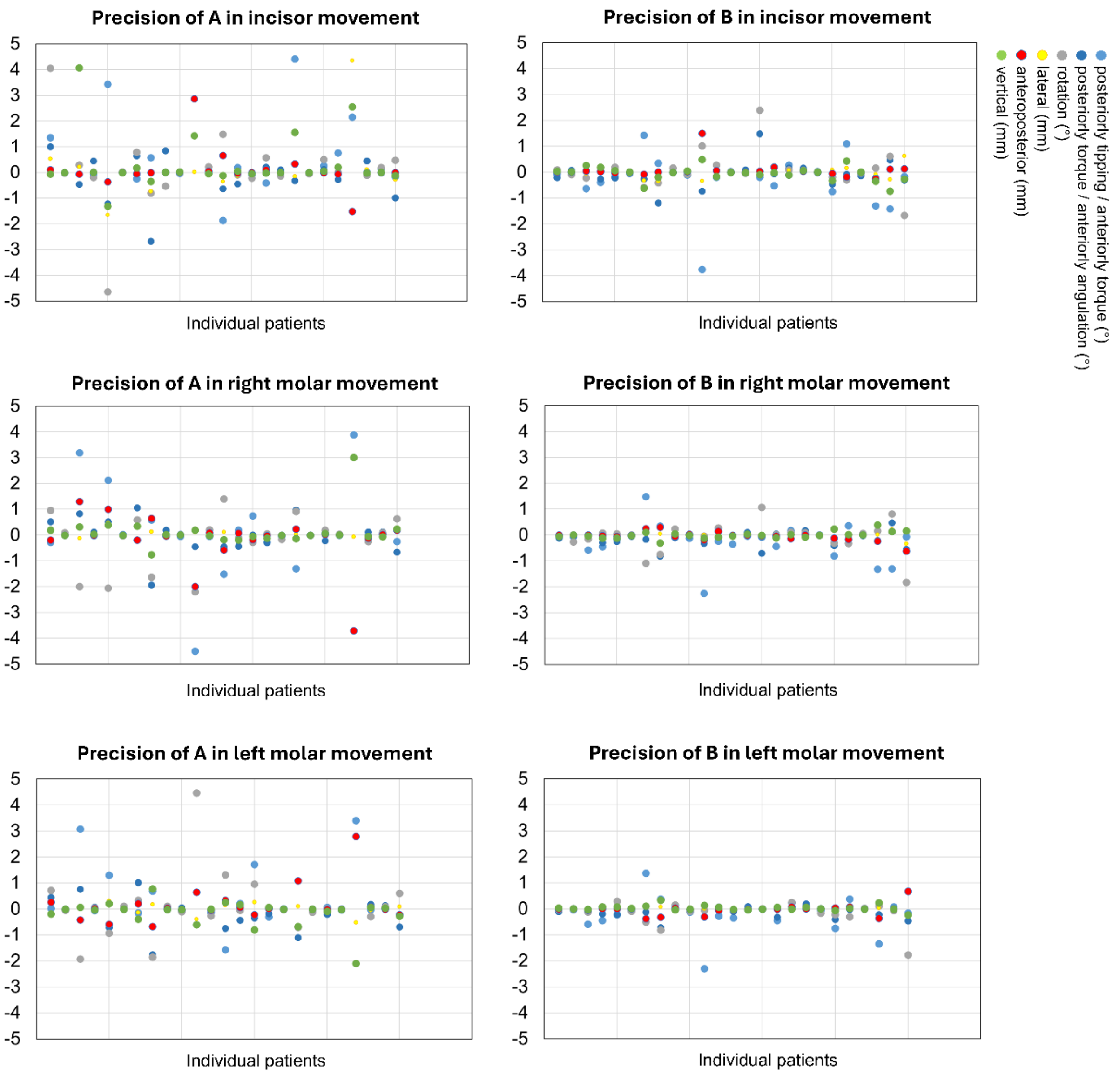

Supplementary Figure 1. Scatter dot plots showing individual precision measurements for each assessed tooth separately, following superimposition on palatal reference areas A and B.

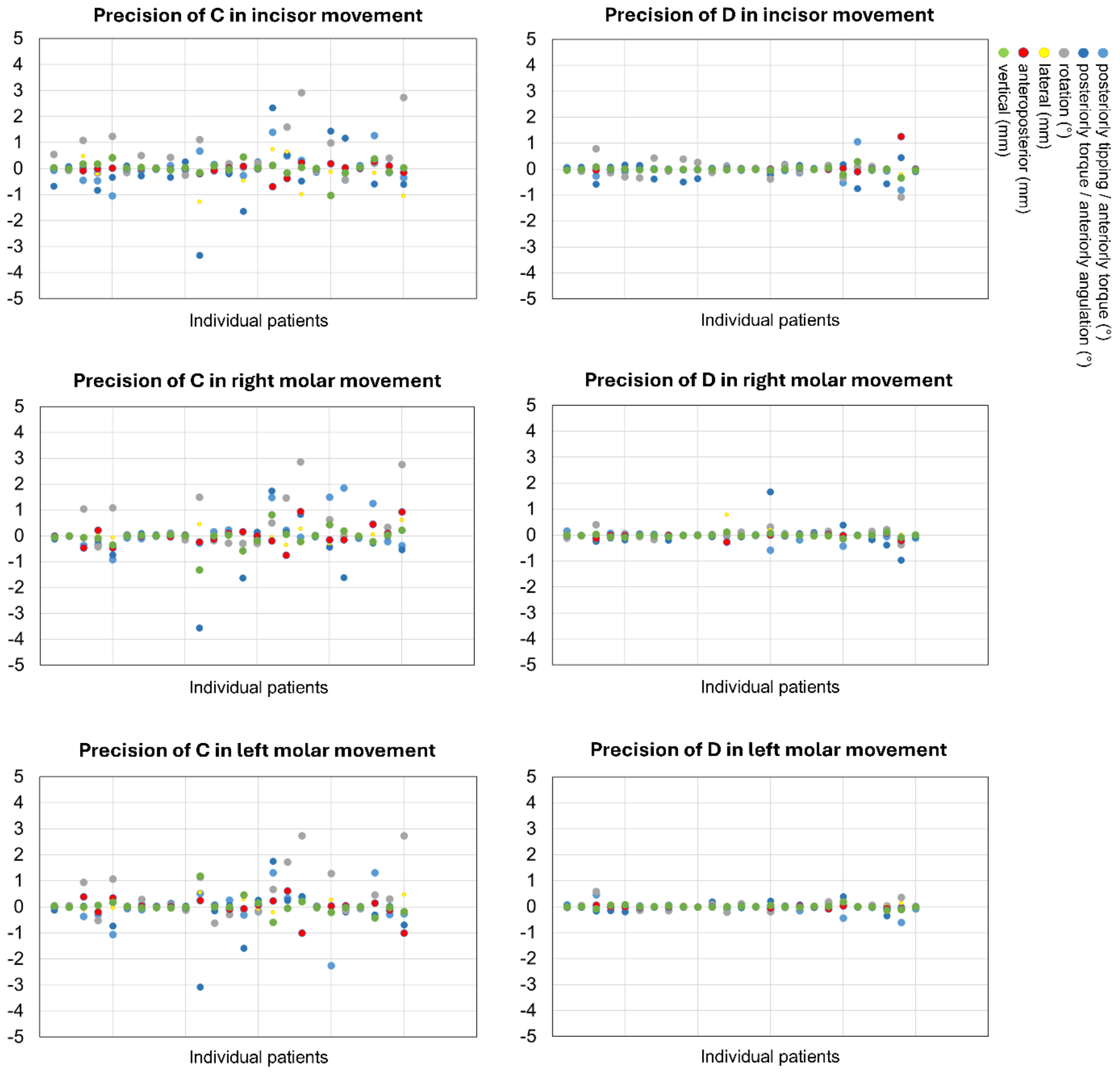

Supplementary Figure 2. Scatter dot plots showing individual precision measurements for each assessed tooth separately, following superimposition on palatal reference areas C and D.
